# Case Report: Two Chinese Infants of Sengers Syndrome Caused by Mutations in *AGK* Gene

**DOI:** 10.3389/fped.2021.639687

**Published:** 2021-06-07

**Authors:** Benzhen Wang, Zhanhui Du, Guangsong Shan, Chuanzhu Yan, Victor Wei Zhang, Zipu Li

**Affiliations:** ^1^Qingdao Women and Children's Hospital, Cheeloo College of Medicine, Shandong University, Jinan, China; ^2^Heart Center, Qingdao Women and Children's Hospital, Affiliated to Qingdao University, Qingdao, China; ^3^Department of Neurology, Qilu Hospital of Shandong University, Jinan, China; ^4^Department of Human and Molecular Genetics, Baylor College of Medicine, Houston, TX, United States

**Keywords:** Sengers syndrome, acylglycerol kinase, mutation, genotype, cardiomyopathy, hypertrophic

## Abstract

Sengers syndrome (OMIM #212350) is a rare autosomal recessive disorder due to mutations in acylglycerol kinase (*AGK*) gene. We report two cases that were diagnosed clinically and confirmed genetically. Both infants had typical clinical features characterized by hypertrophic cardiomyopathy, bilateral cataracts, myopathy, and lactic acidosis, and heart failure was the most severe manifestation. Genetic testing of a boy revealed a homozygous pathogenic variant for Sengers syndrome in *AGK* (c.1131+2T>C) which was classified as likely pathogenic according to the ACMG guideline; besides, his skeletal muscle biopsy and transmission electron microscope presented obvious abnormity. One girl had compound heterozygous (c.409C>T and c.390G>A) variants of *AGK* gene that was identified in the proband and further Sanger sequencing indicated that the parents carried a single heterozygous mutation each. After the administration of “cocktail” therapy including coenzyme Q10, carnitine, and vitamin B complex, as well as ACEI, heart failure and myopathy of the boy were significantly improved and the condition was stable after 1-year follow-up, while the cardiomyopathy of the girl is not progressive but the plasma lactate acid increased significantly. We present the first report of two infants with Sengers syndrome diagnosed via exome sequencing in China.

## Introduction

Sengers syndrome (OMIM #212350) is a rare autosomal recessive disorder due to mutations in the acylglycerol kinase (*AGK*) gene (1). The disease was first described by Sengers in 1975 with the hallmark signs of hypertrophic cardiomyopathy, congenital cataract, mitochondrial myopathy, and lactic acidosis after exercise and can be further divided into two clinical forms including a severe neonatal form that can cause infantile death and a benign form with better prognosis (1, 2). Nystagmus, esotropia, eosinophilia, and cervical meningocele are relatively rare clinical manifestations (3, 4).

The *AGK* gene is located on chromosome 7q34 and consists of 16 exons (1). To date, several studies have identified different types of loss-of-function mutations in the *AGK* gene, including start codon mutations, nonsense, frameshift, and splice site mutations (1–3, 5–16). AGK is a mitochondrial protein that catalyzes the phosphorylation of diacylglycerol (DAG) and monoacylglycerol (MAG) to phosphatidic acid (PA) and lysophosphatidic acid (LPA), respectively. PA feeds into the synthesis of the mitochondrion-specific lipid cardiolipin (CL), which is essential for mitochondrial structure and function (2).

Sengers syndrome is often misdiagnosed due to its rarity. Currently, <40 cases diagnosed by genetic testing have been reported in the literature although other cases may not have been reported and the prevalence remained unclear. There is no cure for this disease and its clinical features and prognosis are also still unclear. We present the clinical characteristics and molecular basis of Sengers syndrome in two Chinese children, the first reported cases in China.

## Case Presentation

### Patient 1

**Figure 1:**
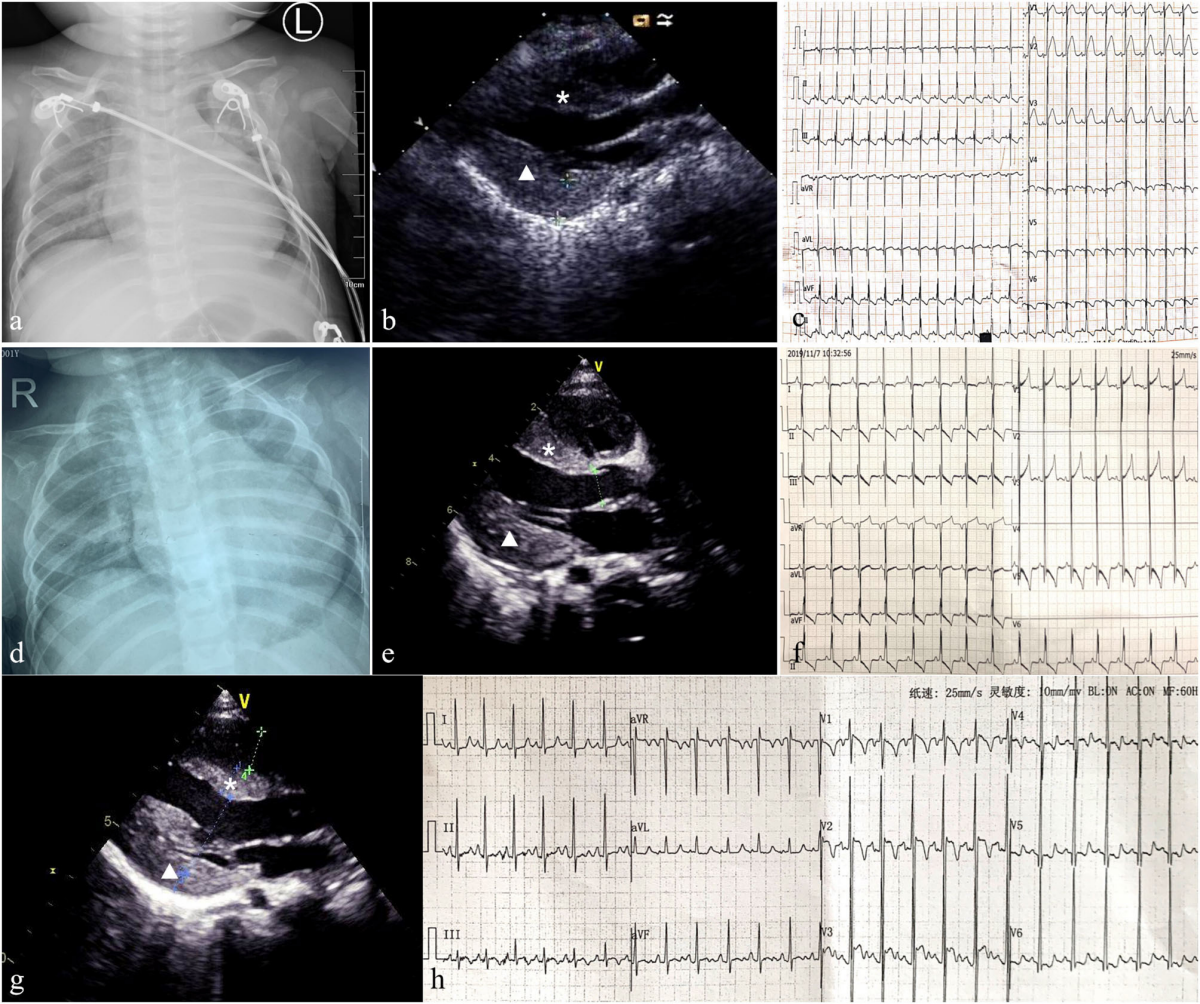
Patient 1: Chest X-ray showed an enlarged heart (the cardiothoracic ratio reached about 0.65) **(a)** and echocardiography indicated hypertrophic non-obstructive cardiomyopathy **(b)**. ECG showed sinus tachycardia, short PR interval, biventricular hypertrophy, and multi-lead ST segment changes **(c)**. Follow-up for 8 months, chest X-ray indicated the CTR was 0.65 **(d)**. Echocardiography showed the thickness of IVS and LVPW were 9 and 11 mm, respectively, with no outflow obstruction, and LVEF was 60% **(e)**. ECG revealed multiple lead ST-T changes and left ventricular hypertrophy **(f)**. Patient 2: Echocardiography showed the thickness of interventricular septum (IVS) and left ventricular posterior wall (LVPW) were 9 and 10 mm, respectively **(g)**. ECG revealed left ventricular hypertrophy and multiple lead ST-T changes **(h)**. The asterisk represents IVS and triangle represents LVPW.

This infant boy was the second child of healthy consanguineous parents from China. After an uneventful pregnancy, he was born at term by vaginal delivery, with a birth weight of 3,260 g, and 10 years after the first sibling. The Apgar score was 9, 10, 10 at 1, 5, 10 min, respectively. The boy was healthy during the first 2 months of life, and he had a mild motor developmental delay characterized by a disability of sitting without support at 7 months of age. His cognitive condition was normal. Bilateral total cataracts were noticed when he was 1 month old. Cataract phacoemulsification and vitrectomy of both eyes were performed at 3 months of age and aphakia was corrected with glasses and intraocular lens implantation was scheduled. The boy was coughing and had inspiratory stridor for 6 days at the age of 8 months of age and was admitted to our hospital. Physical examination showed no obvious dysmorphic features, no rash, shortness of breath, a rapid heart rate of 180 bpm, no heart murmur, and moderate hepatomegaly. Moreover, severe hypotonia and decreased muscle strength were observed (grade 2 of MRC 6-point scale). The chest X-ray showed an enlarged heart, the cardiothoracic ratio (CTR) reached about 0.65 (Figure 1a). Echocardiography showed hypertrophic cardiomyopathy with a maximum septal thickness of 8 mm, a posterior wall thickness of 8 mm, no outflow tract obstruction, enlargement of left ventricle, and ejection fraction of 39% (Figure 1b). Electrocardiogram showed sinus tachycardia, short PR interval (100 ms), biventricular hypertrophy, and multi-lead ST-segment changes (Figure 1c). Arterial blood gas results were relatively normal except for mild lactic acidosis (lactate 3.7 mmol/L, reference ranges: lactate <2.0 mmol/L). Other laboratory analyses were normal which included blood routine testing, routine urine testing, alanine transaminase, aspartate aminotransferase, creatinine, urea nitrogen, blood glucose, and blood ammonia levels. N-terminal pro-brain natriuretic peptide (NT-proBNP) reached 6,076 pg/ml (reference ranges <125 pg/ml) reflecting the state of the heart failure. Further metabolic work-up, such as urinary organic acids and amino acids, blood carnitine, and acid alpha-glucosidase tests were also normal.

**Figure 2:**
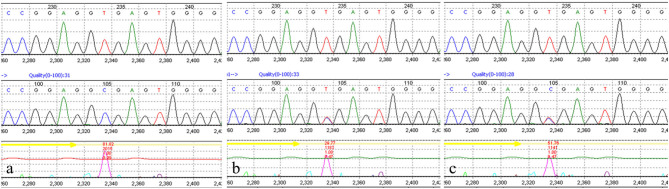
Molecular study of the genomic DNA of patient 1 detected a novel AGK homozygous mutation (c.1131 + 2T>C) in the proband **(a)**. Both parents were confirmed to be heterozygous carriers **(b,c)**.

The medical exome of the proband was enriched before sequencing which included about 4,200 known disease genes as well as known pathogenic variants located in deep intronic and other non-coding regions. After genetic analysis, a novel homozygous (c.1131+2T>C) variant of *AGK* gene was identified in the proband. Both parents were unaffected and further Sanger sequencing indicated that both the parents were heterozygous for this change (Figure 2). The c.1131+2T>C variant in the *AGK* gene has not been previously reported in clinical cases. However, defects in *AGK* gene have been reported to be associated with Sengers syndrome. The clinical presentation includes congenital cataracts, hypertrophic cardiomyopathy, skeletal myopathy, exercise intolerance, and lactic acidosis. Mental development is normal, but affected individuals may die early from cardiomyopathy (2). Skeletal muscle biopsies of two affected individuals showed severe mtDNA depletion (8). Altogether, *AGK* gene was identified as the causative gene in the proband and the variant c.1131+2T>C was classified as likely pathogenic according to the American College of Medical Genetics and Genomics (ACMG) guidelines.

**Figure 3:**
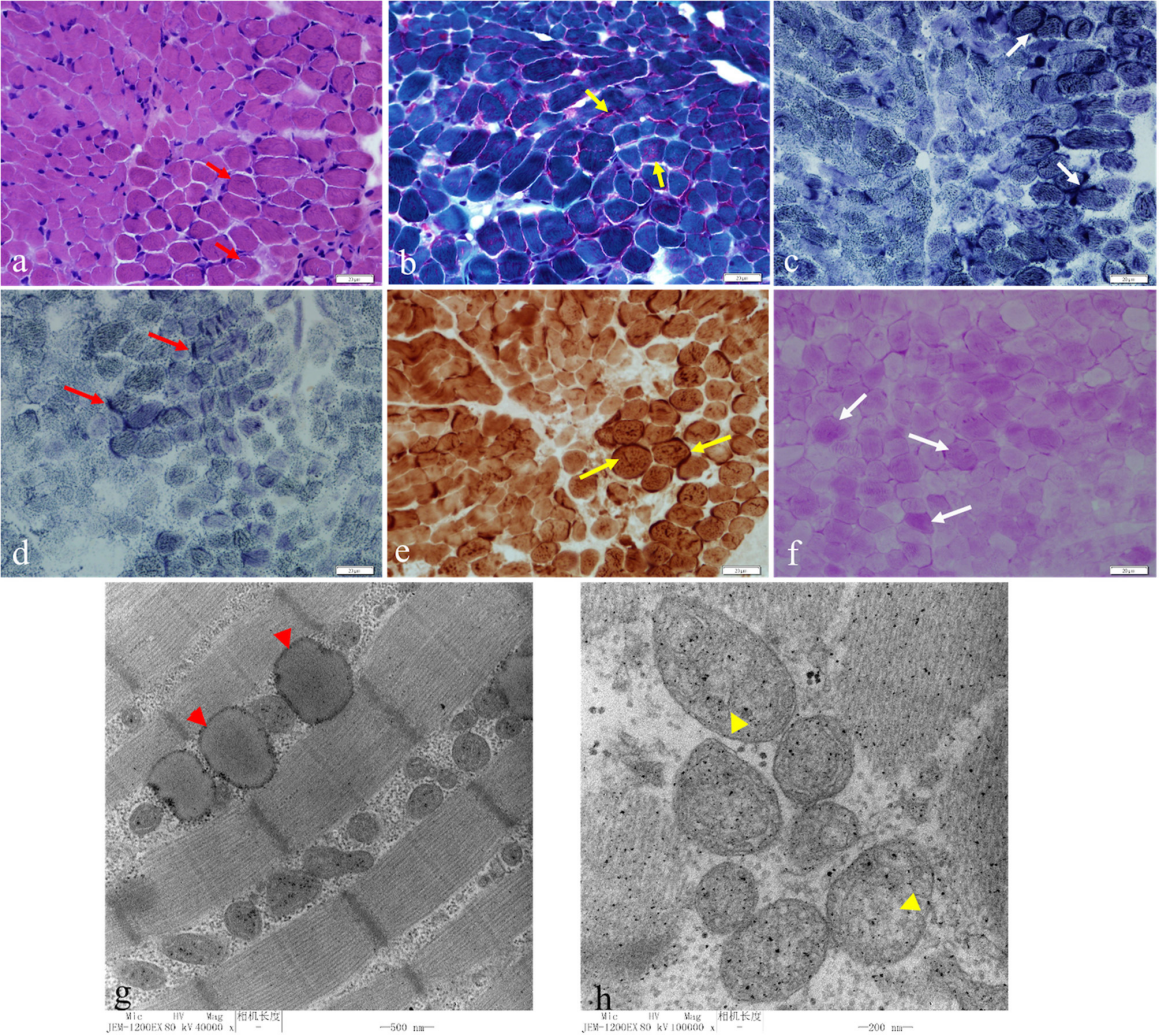
HE staining showed that the muscle fibers were slightly different in size, and the small fibers were mostly small round and polygonal in shape. Cavitation and fissures were observed in the fibers with most fibers deeply staining the sarcolemma **(a)** (red short arrow, bar 20 μm). Modified Gomeri tricolor (MGT) staining displayed a large number of muscle fibers showing cytoplasmic and sub-sarcolemma vacuole fissures with RRF-like changes **(b)** (yellow short arrow, bar 20 μm). Deep staining was seen under the sarcolemma in many fibers in nicotinamide adenine dinucleotide (NAD) **(c)** (white short arrow, bar 20 μm), succinate dehydrogenase (SDH) **(d)** (red long arrow, bar 20 μm), cytochrome *c* oxidase (COX) staining **(e)** (yellow long arrow, bar 20 μm). The glycogen content of a small number fibers increased significantly with periodic acid Schiff (PAS) staining **(f)** (white long arrow, bar 20 μm). Transmission electron microscopy (TEM) showed an increased number of lipid droplets in many fibers **(g)** (red arrowhead, bar 500 nm) and the mitochondria were abnormal with a severe loss of cristae **(h)** (yellow arrowhead, bar 200 nm).

A muscle biopsy was performed revealing obvious structural changes. HE stain showed that the muscle fibers were slightly different in size, and the small fibers were mostly small round and polygonal in shape (Figure 3a). Cavitation and fissures were observed in the fibers. Moreover, most fibers had deeply stained sarcolemma. Modified Gomeri tricolor (MGT) stain displayed a large number of muscle fibers showing cytoplasmic and sub-sarcolemma vacuole fissures with RRF-like changes (Figure 3b). Deep staining was observed for sarcolemma in many fibers with nicotinamide adenine dinucleotide (NAD), succinate dehydrogenase (SDH), and cytochrome *c* oxidase (COX) staining (Figures 3c–e). Furthermore, the content of glycogen of a small number of fibers increased significantly with periodic acid Schiff (PAS) staining and the content of lipid droplets in a large number of fibers markedly increased in oil red (ORO) staining (Figure 3f). Transmission electron microscope (TEM) revealed an increased number of lipid droplets in many fibers and the mitochondria were abnormal with a severe loss of cristae (Figures 3g,h).

Supplementation with coenzyme Q10, carnitine, B-vitamins, and biotin (called mitochondrial cocktail) was given daily, associated with angiotensin converting enzyme (ACE) inhibitors for cardiomyopathy management. The patient received milrinone, diuretics (furosemide and spironolactone), and captopril to improve heart function. Levocarnitine (100 mg/kg daily), coenzyme Q10 (1 mg/kg daily), and vitamin B complex (vitamin B_1_ 20 mg/day and riboflavin 10 mg/day) were administered to improve metabolic status; however, anti-infection and other symptomatic treatments were also applied. After 10 days of treatment, respiratory symptoms were alleviated, and muscle strength improved to grade 5 of MRC 6-point scale. Echocardiography showed no significant improvement in myocardial hypertrophy(septal thickness of 9 mm, a posterior wall thickness of 10 mm), left ventricular end-diastolic dimension (LVEDd) reduced from 33 to 23 mm, and heart function returned to normal (EF of 67%). Contrary to clinical improvement, plasma lactate acid rose to 13 mmol/L. Milrinone was stopped and the remaining oral drugs continued to be used. To date, this child has been followed up for 8 months and is 18 months of age. He had no recurrent respiratory infections, his height and body weight were 75 cm and 8 kg, respectively, and no cognition delay was detected. Mild motor retardation existed which was characterized by walking utilizing support. Physical examination showed no tachypnea, an average heart rate of 100 bpm, lack of cardiac positive signs, no liver enlargement, no hypotonia, and muscle strength grade 5. Plasma lactate acid was 3.1 mmol/L. The chest X-ray indicated that the CTR was 0.65 (Figure 1d). Echocardiography showed a septal thickness of 9 mm, a posterior wall thickness of 11 mm, and LVEF of 66% (Figure 1e). ECG revealed multiple lead ST-T changes and left ventricular hypertrophy (Figure 1f).

### Patient 2

A girl was the third child born by normal delivery at full term after a normal pregnancy with a birth weight of 2250 g. His older sister died of “brain herniation” at 4 months old, and his older brother is alive and healthy. The newborn had no obvious muscle hypotonia. At age 2 months, bilateral cataracts were noted and cataract phacoemulsification was performed at 3 months of age. In the following 3 months, the patient developed growth retardation; physical examination showed no obvious dysmorphic features, no heart murmur, no hepatomegaly, no hypotonia, and decreased muscle strength was observed (grade 4 of MRC 6-point scale). HCM was detected that echocardiography revealed a septal thickness of 9.1 mm and posterior wall thickness of 9.5 mm without the presence of an obstructive component, and electromyographic activity was weak. Blood gas analysis revealed metabolic acidosis with elevated serum lactic acid (4.1 mmol/L, normal <2.0 mmol/L). Urinary organic acid analysis showed increased amounts of 3-hydroxybutyrate (25.5 mmol/L, normal <9.0 mmol/L) and NT-proBNP reached 253 pg/ml. Laboratory studies in serum yielded normal results for the following: hematological parameters, electrolytes, liver function, renal function, creatine kinase, cholesterol, triglycerides, glucose, and ammonia levels.

The medical exome of the proband was used for genetic analysis, compound heterozygous (c.409C>T and c.390G>A) variants of *AGK* gene were identified in the proband, and further Sanger sequencing indicated that the parents carried a single heterozygous mutation each. The nonsense c.409C>T variant in the *AGK* gene has already been described at a homozygous state in several cases which had typical Sengers syndrome manifestations. The splicing c.390G>A variant in the *AGK* gene has not been previously reported in clinical cases and was classified as likely pathogenic according to the ACMG guidelines by genetic evaluation of pathogenicity of variants using multiple computational algorithms. Currently, this patient has been treated with “mitochondrial cocktail,” ACEI, and beta-blockers for more than 1 year. She has significant growth retardation and moderate muscle weakness, especially in the lower limb muscles, and she can just keep a standing position with help. The good part is that the cardiomyopathy is not progressive which echocardiography showed a septal thickness of 9 mm and posterior wall thickness of 10 mm (Figure 1g). ECG revealed left ventricular hypertrophy and multiple lead ST-T changes (Figure 1h). However, the serum lactic acid increased significantly, reaching 14.99 mmol/L, so appropriate limitation of physical activities is recommended in daily life.

## Discussion

Sengers syndrome is a rare mitochondrial disease caused by mutations in the *AGK* gene (4). To date, the incidence of this disease is difficult to estimate and the cases in our study are the first cases reported in China. AGK, also known as multi-substrate lipid kinase (MULK), affects the synthesis of phosphatidic acid which acts as a second messenger regulating a number of cellular processes and plays an important role in the synthesis of phospholipids (1). Many studies found oxidative phosphorylation (OXPHOS) defects in Sengers syndrome and suggested that mitochondrial respiration and metabolism are affected in the absence of AGK (1, 3, 7). Kang et al. suggested that AGK is a subunit of the mitochondrial TIM22 protein import complex where it facilitates the import and assembly of mitochondrial carrier proteins. Furthermore, the TIM22 complex and carrier import was demonstrated to be affected in Sengers syndrome cells and tissues (5). These findings showed that the role of AGK in Sengers syndrome patients may explain the characteristics of mitochondrial morphology, cataracts, and respiratory chain dysfunction.

Before the widespread application of genetic testing and the determination of pathogenic mutations in *AGK* gene, the diagnosis of Sengers syndrome mainly relied on characteristic clinical manifestations. Therefore, some studies have reported some confirmed or suspicious cases without molecular diagnostic evidence (17–19). Herein, we reviewed previous studies on this syndrome and summarize the characteristics of the cases with both clinical and genetic information (Table 1, Supplementary Material 1). Thus far, a total of 38 children have been diagnosed with the confirmation of gene test, of which 60% were males. In terms of mortality, Sengers syndrome is a highly malignant disease, with a total mortality rate of 57.9% (22/38). Importantly, the death rate varied at different ages, namely, 86.4% (19/22) within 3 years of age, 77.3% (17/22) within 1 year of age, and 31.8% (7/22) during the neonatal period. Concerning the clinical manifestations, the overwhelming majority (26/38, 68.4%) of the patients had their features in the neonatal period. Cataracts were the most common clinical manifestation (94.7%), followed by hypertrophic cardiomyopathy (65.8%), lactic acidemia (71.1%), and myopathy (65.8%). Other rare clinical manifestations included nervous system issues, such as cerebellar non-hemorrhagic stroke, and ocular signs, such as nystagmus. The most fatal features were cardiac failure and cardiac arrest.

**Table 1:** Clinical and molecular findings in patients with Sengers-syndrome caused by AGK mutations.

**Case**	**Described by**	**Gender**	**Mutations**		**Onset of disease**	**Cardiomyopathy**	**Cataract**	**Myopathy**	**LA**	**Other presentation**	**Biopsy**	**OXPHOS defect**	**Status**
			**Allele 1**	**Allele 2**									
1	Present case 1	M	c.1131+2T>C, Splicing defect	N	8m	+	+	+	+	–	+	NA	Alive 2 y
2	Present case 2	F	c.409C>T, p.Arg137*	c.390G>A, p.Glu130Glu	2m	+	+	+	+	–	–	NA	Alive 2.5 y
3	Elliott et al. (9)	F	c.409C>T, p.Arg137*	c.841C>T, p.Arg281*	1d	+	+	NA	NA		NA	NA	NA
4	Lalive et al. (10)	M	c.3G>C, p.Met1Ile	c.517C>T, p.Gln173*	3m	+	+	+	+		NA	NA	Alive 6 y
5	Lalive et al. (10)	M	c.3G>C, p.Met1Ile	c.672C>A, p.Tyr224*	3m	+	+	+	+		NA	NA	Alive 35 y
6	Morava et al. (4)	M	c.1131+5G>A, Splicing defect	N	18m	+	+	–	+		+	I, II + III, IV, V, PDHc	Dead 12 y
7	Morava et al. (4)	F	c.1131+5G>A, Splicing defect	N	5m	+	+	+	+		+	I, II + III, IV, V, PDHc	Alive 10 y
8	Van Ekeren et al. (12)	F	c.1131+5G>A, Splicing defect	N	Birth	+	+	+	+	Stroke, cerebrovascular accident		V	Alive 41 y
9	Rosa et al. (12)	F	c.221+1G>A, Splicing defect	c.1213C>T, p.Gln405*	Birth	+	+	+	+		+	–	Alive 12 y
10	Mayr et al. (2)	M	c.306C>T, p.Tyr102*	c.841C>T, p.Arg281*	Birth	+	+	–	+	Floppy infant	+	I, II+III, IV, V	Dead 18 d
11	Mayr et al. (2)	F	c.672C>A, p.Tyr224*	c.870del, p.Gln291Argfs*8	Birth	+	+	+	+	Cardiac arrest	+		Dead 10 m
12	Mayr et al. (2)	M	c.101+?222–?del	N	4m	+	+	+	+	Seizures, upper left limb paresis, brain ventricles dilatation	+		Dead 8 m
13	Mayr et al. (2)	M	c.412C>T, p.Arg138*	c.1137_1143del, p.Gly380Leufs*16	1w	+	+	+	+		+	I	Dead 11 m
14	Haghighi et al. (1)	M	c.523_524del#p.Ile175Tyrfs*2	N	1m	+	+	+	+	Nystagmus, floppy infant	NA	NA	Dead 7 m
15	Haghighi et al. (1)	M	c.424-1G>A, Splicing defect	N	Birth	+	+	–	+		NA	NA	Dead 10 d
16	Haghighi et al. (1)	F	c.424-1G>A, Splicing defect	N	Birth	+	+	–	+	Eosinophilia	NA	NA	Dead 4 m
17	Haghighi et al. (1)	F	c.409C>T, p.Arg137*	N	NA	+	+	–	+	Esotropia	Fatty infiltrations (heart)	I	Dead 3 m
18	Haghighi et al. (1)	M	c.409C>T, p.Arg137*	N	Birth	+	+	+	+	Esotropia, nystagmus, floppy infant	+	I	Dead 6 m
19	Haghighi et al. (1)	M	c.871C>T, p.Gln291*	c.1035dup, p.Ile346Tyrfs*39	Birth	+	+	+	+			NA	Alive 3 m
20	Haghighi et al. (1)	F	c.297+2T>C, p.Lys75Glnfs*12	c.841C>T, p.Arg281*	Birth	+	+	+	–	Cervical meningocele, language delay		I	Alive 10 y
21	Haghighi et al. (1)	M	c.877+3G>T, Splicing defect	N	Birth	+	+	+	–			NA	Alive 15 y
22	Calvo et al. (8)	F	c.297+2T>C, p.Lys75Glnfs*12	c.1170T>A, p.Tyr390*	<1y	+	+	+	+	Headaches, osteopenia, premature ovarian failure	+	I, III, IV	Dead 18 y
23	Calvo et al. (8)	F	c.1131+1G>T, p.Ser350Glufs*19	N	Birth	+	+	–	+		+	I, III, IV	Dead 4 d
24	Siriwardena et al. (3)	F	c.979A>T, p.Lys327*	N	Birth	+	+	+	NA	Cardiac failure		NA	Dead 5 m
25	Siriwardena et al. (3)	F	c.979A>T, p.Lys327*		Birth	+	+	–	–	Upper respiratory tract infection		I, I + III, II + III, III, IV, high CS	Dead 12 d
26	Siriwardena et al. (3)	M	c.979A>T, p.Lys327*	N	Birth	NA	NA	NA	NA		NA	NA	Dead 2 d
27	Siriwardena et al. (3)	M	c.979A>T, p.Lys327*	N	Birth	NA	NA	NA	NA		NA	NA	Dead 18 d
28	Siriwardena et al. (3)	M	c.3G>A, p.Met1?(p.M1I)	N	9m	+	+	+	+	Cerebellar non-hemorrhagic stroke, cardiac arrest, ventricular fibrillation	Scattered COX negative fibers	NA	Dead 15 m
29	Siriwardena et al. (3)	M	c.3G>A, p.Met1?(p.M1I)	N	Birth	+	+	+	+	Cerebellar non-hemorrhagic stroke	–	NA	Alive 2.5 y
30	Aldahmesh et al. (7)	F	c.424-3C>G, p.Ala142Thrfs*4	N	Birth	–	+	–	–		NA	NA	Alive 17 y
31	Aldahmesh et al. (7)	M	c.424-3C>G, p.Ala142Thrfs*4	N	Birth	–	+	–	–		NA	NA	Alive 11 y
32	Aldahmesh et al. (7)	M	c.424-3C>G, p.Ala142Thrfs*4	N	Birth	–	+	–	–		NA	NA	Alive 7 y
33	Kor et al. (13)	M	c.297G>T, p.K99N	N	5d	+	+	+	+	Cardiac failure	Fatty infiltrations	NA	Dead 22 m
34	Kor et al. (13)	F	c.412C>T, p.R138*	N	1m	+	+	+	+	Cardiac failure	NA	NA	Dead 3 m
35	Allali et al. (14)	M	c.1035dup, p.Ile346Tyrfs*39	N	3m	+	+	+	+	Macrocephaly, cognitive deficiency, nystagmus	NA	NA	Alive 9 y
36	Allali et al. (14)	M	c.1035dup, p.Ile346Tyrfs*39	N	Birth	+	+	+	NA	Phenylketonuria, nystagmus, language delay	NA	NA	Dead 2 y
37	Beck et al. (15)	M	c.979A >T, p.K327*	N	Birth	+	+	+	+	Chorioamniotis, hepatic dysfunction	NA	NA	Dead 1 d
38	Guleray et al. (16)	F	c.1215dupG, p.Phe406Valfs4	N	3m	+	+	+	+	–	Lipid deposition Decreased COX Staining (heart and liver)	NA	Dead 9 m

*M, male; F, female; N, none; NA, not available; LA, lactic acidosis; OXPHOS, oxidative phosphorylation; +, presence of condition; –, absence of condition; unknown when empty; I, complex I; II + III, succinate cytochrome c oxidoreductase; IV, cytochrome c oxidase; V, oligomycin-sensitive ATPase; PDHc, pyruvate dehydrogenase complex; d, day; m, month; y, year*.

Considering the typical clinical manifestations of Sengers syndrome, we need to differentiate it from syndromes characterized by hypertrophic cardiomyopathy, muscle weakness, and growth retardation, such as RASopathy disorder and Pompe disease (20, 21). These syndromes often have hypertrophic cardiomyopathy as the primary clinical feature, and muscle weakness can also be easily found through physical examination. In particular, we should note that in the case of combined infection, worsening heart failure, etc., severe hyperlactatemia can also occur in the aforementioned diseases. Certainly, there are also some special points of differential diagnosis. Patients with RASopathy disorder often have more typical facial abnormalities, and congenital heart diseases, such as pulmonary valve stenosis and ventricular septal defect, are more common (20). Pompe disease and other inherited metabolic diseases often have a definite enzymatic deficiency, and the test methods are efficient and convenient to facilitate early diagnosis. The main point of distinguishing Sengers syndrome from the above diseases is ocular lesions. However, due to the early onset of Sengers syndrome, the condition of ocular involvement may be missed, resulting in misdiagnosis or delay in diagnosis.

Given that Sengers syndrome is a very rare genetic disorder, the genotype/phenotype correlation has remained unclear. The severity of the disease is dictated by the combination of the two alleles, and so a child homozygous for a more deleterious mutation (e.g., a nonsense mutation) or compound heterozygous for two severe deleterious mutations may be expected to have earlier mortality; a child homozygous for a less deleterious mutation may have longer survival. This result is consistent with previous reports that homozygous *AGK* nonsense mutations have resulted in a severe form of Sengers syndrome (1). Aldahmesh et al. identified a splice site mutation causing isolated congenital cataracts in three patients, and it can be speculated that a small proportion of the normally spliced transcript can still be formed (1, 7).

Genetic investigations confirmed that one of our patients has Sengers syndrome due to a novel homozygous variant in the *AGK* gene which the splicing algorithm (splice-port) predicted to affect the splice donor site of intron 15 (c.1131+2T>C). This site is highly conserved across species and this mutation has not been reported in clinical cases. Moreover, other software programs predict that it may affect splicing. Combined with the typical clinical manifestations, pathological changes of the child, we classified this variant as pathogenic or mutation. Three individuals from two more families from the Netherlands harbored homozygous mutations near the c.1131 site which the splice-port algorithm predicted to affect the splice donor site of intron 16 (c.1131+5G>A) (2). Cardiomyopathy, cataract, and lactic acidosis were common manifestations in all three patients, which is consistent with our study. OXPHOS defects were detected in two patients; only one patient died (death at 12 years old), and the remaining two patients were reported to be alive at the time of the report (42 and 10 years of age) (2). Overall, these patients had a relatively long lifespan, and this may suggest a better prognosis for intron splicing mutations near c.1131 area. The nonsense c.409C>T variant in the *AGK* gene has already been described at a homozygous state in several typical Sengers syndrome cases, and this suggests that the gene is pathogenic (1). According to the clinical manifestations and the results of genetic analysis, the splicing c.390G>A variant is likely to be pathogenic, and further evidence needs to be accumulated. However, the clinical manifestations are different among the reported cases. It may be plausible that the characteristics may be associated with genotype. For instance, *AGK* homozygous nonsense mutations are common in critical patients which can develop cardiomyopathy and fatal lactic acidosis in infancy (1, 2, 4).

There is currently no curative therapy for mitochondrial disorders, although symptomatic measures can be highly effective and greatly improve the quality of life and outcomes for these patients (22). According to current investigations and the small population with Sengers syndrome, there is no specific treatment strategy for this disease. In our study, following the administration of anti-heart failure drugs and “cocktail” therapy, the boy's heart failure, myopathy, and other clinical features significantly improved and the girl's cardiomyopathy is not progressive. This result suggested that symptomatic treatment has a significant effect on this disease, and the clinical outcome from cardiac side is likely related to anti-failure drugs. Treatment for energy metabolism may be also an effective strategy for ameliorating respiratory chain disorders; however, it should also be noted that the “cocktail” treatment is not backed up by evidence yet, although common in clinical practice and unlikely harmful. In addition, drug therapy may not be a need for the lactic acidosis seen in Sengers syndrome, and limiting the amount of exercise may have a role in the controlling of hyperlactatemia.

## Data Availability Statement

The datasets presented in this study can be found in online repositories. The names of the repository/repositories and accession number(s) can be found at: https://www.ncbi.nlm.nih.gov/clinvar, VCV000987519; VCV000987518; VCV000209130.

## Ethics Statement

The studies involving human participants were reviewed and approved by the ethics committee of Qingdao Women and Children's Hospital. Written informed consent to participate in this study was provided by the participants' legal guardian/next of kin.

## Author Contributions

BW was responsible for interpretation of the data, drafting of the article, and approval of the final version to be published. ZD was responsible for interpretation of the data and drafting of the article. GS was responsible for data collection. CY's laboratory was responsible for specimen processing. ZL was responsible for the study conception, interpretation, revision of the article, and approval of the final version to be published. All authors read and approved the final article.

## Conflict of Interest

The authors declare that the research was conducted in the absence of any commercial or financial relationships that could be construed as a potential conflict of interest.

## Supplementary Material

Supplementary Material 1 Gene structure of AGK and localization of identified mutations (1–3, 5–16). Red font indicates newly reported mutations.

Supplementary Material 2 Normal range for reported laboratory values.

Supplementary Material 3 Sequential echocardiogram results of two cases.
