# Supplementary figures and images for: Case Report: Two Chinese Infants of Sengers Syndrome Caused by Mutations in AGK Gene
Source: Front Pediatr. 2021 Jun 7;9:639687. doi: 10.3389/fped.2021.639687 (PMC8215120; doi:10.3389/fped.2021.639687)

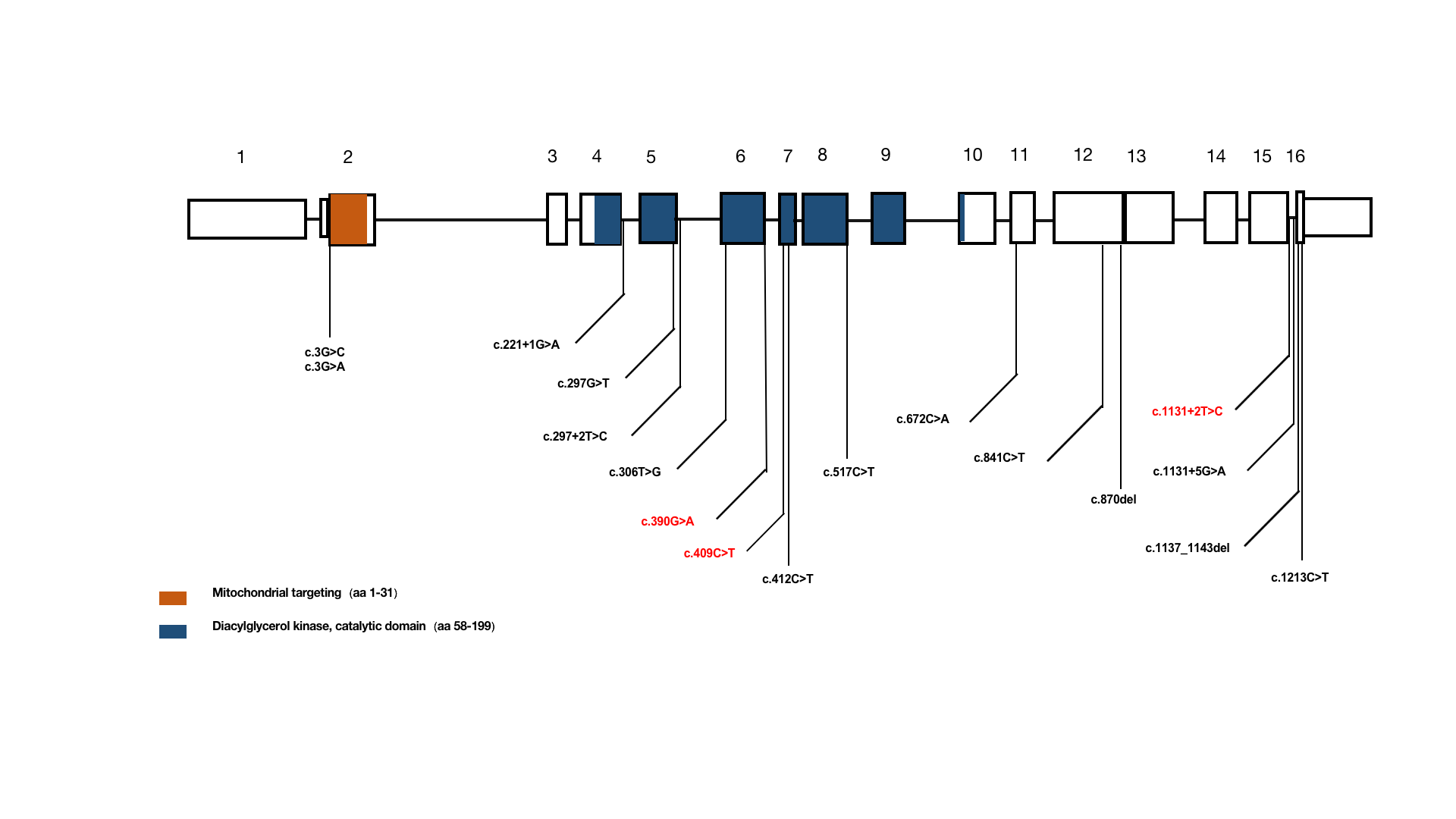

Supplement: Supplementary Material 1 — Gene structure of AGK and localization of identified mutations (1–3, 5–16). Red font indicates newly reported mutations. [file Image_1.TIF]

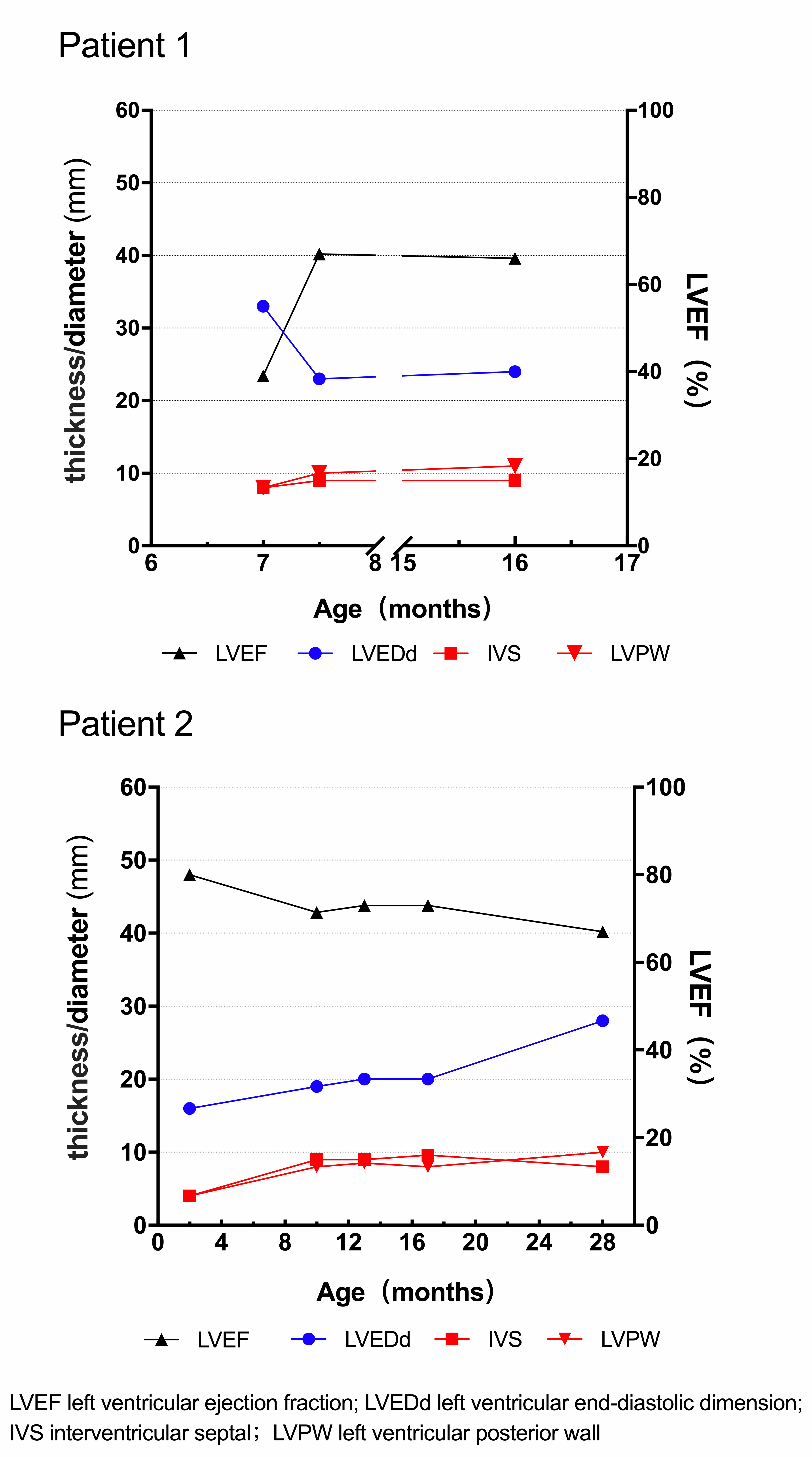

Supplement: Supplementary Material 3 — Sequential echocardiogram results of two cases. [file Image_2.TIFF]
