# Supplementary material for: Case Report: Two Chinese Infants of Sengers Syndrome Caused by Mutations in AGK Gene
Source: Front Pediatr. 2021 Jun 7;9:639687. doi: 10.3389/fped.2021.639687 (PMC8215120; doi:10.3389/fped.2021.639687)
Supplement: Supplementary Material 2 — Normal range for reported laboratory values. [file Data_Sheet_1.PDF]

Supplementary 2 Normal range for reported laboratory values

Items	Range	Unit
lactate	lactate < 2.0	mmol/L
blood routine testing		
white blood cell	4-12	$\times 10^9$/L
platelet	100-300	$\times 10^9$/L
neutrophil ratio	20-40	%
lymphocyte ratio	50-70	%
hemoglobin	110-170	g/L
red blood cell	3.5-5.3	$\times 10^{12}$/L
hematocrit	80-100	%
routine urine testing		
PH	5-8	
glucose	negative	
red cell	negative	/HPF
ketone	negative	
bilirubin	negative	
leucocyte	negative	/HPF
alanine transaminase	7-40	U/L
aspartate aminotransferase	13-40	U/L
creatinine	41-73	umol/L
urea nitrogen	2.6-8.0	mmol/L
creatinine kinase	40-200	U/L
creatinine kinase-MB	0-25	U/L
cholesterol	0-5.18	mmol/L
triglyceride	0-1.7	mmol/L
electrolytes		
potassium	3.5-5.3	mmol/L
sodium	137-147	mmol/L
calcium	2.11-2.52	mmol/L
chlorine	99-110	mmol/L
blood glucose	3.3-5.6	mmol/L
blood ammonia levels		
N-terminal pro-brain natriuretic peptide (NT-proBNP)	<125	pg/ml
acid alpha-glucosidase tests	>14	nmol/1h/mg
